# Supplementary figures and images for: A core of functional complementary bacteria infects oysters in Pacific Oyster Mortality Syndrome
Source: Anim Microbiome. 2023 May 3;5:26. doi: 10.1186/s42523-023-00246-8 (PMC10155333; doi:10.1186/s42523-023-00246-8)

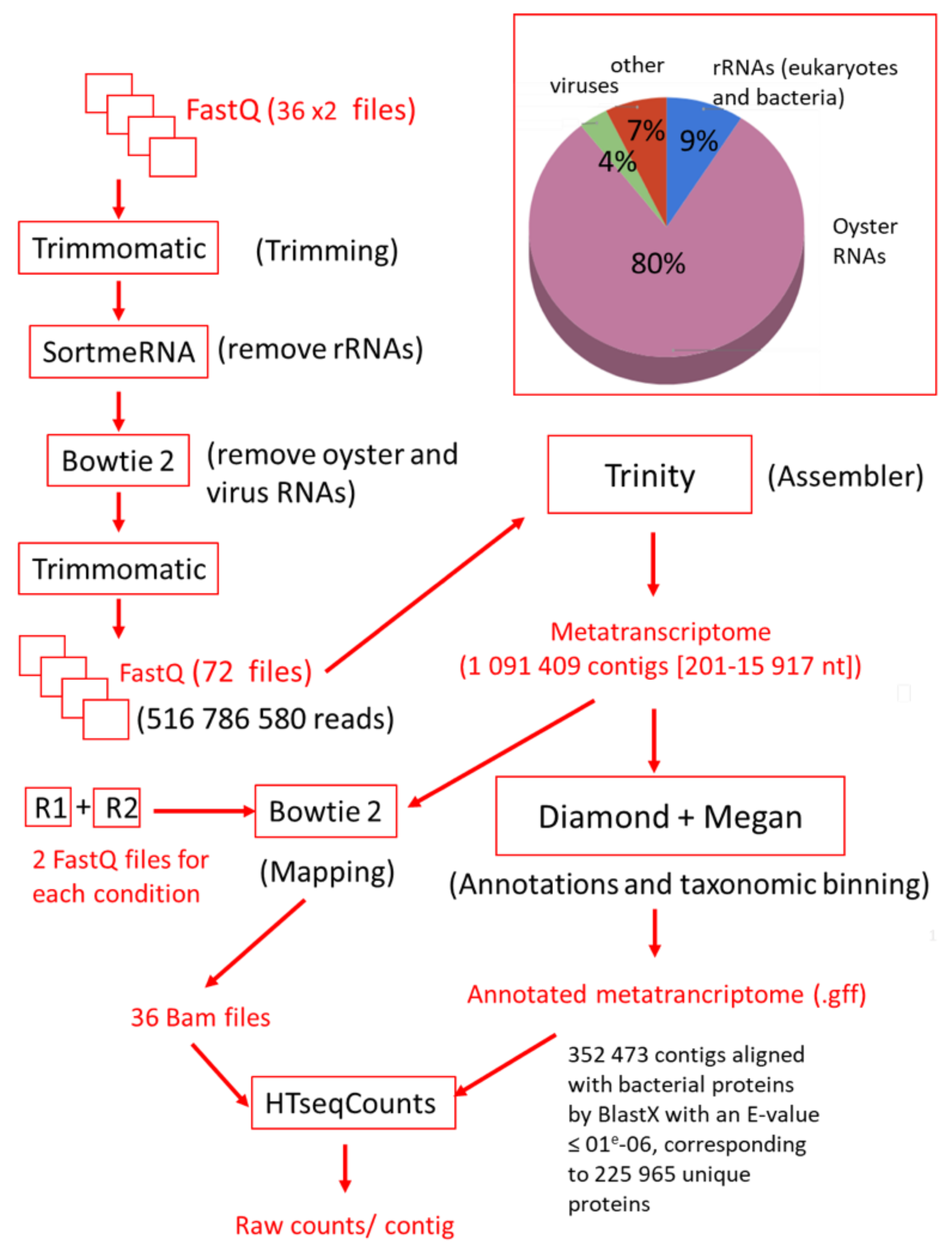

Supplement: Supplementary file 1 — Additional file 1. Figure S1. Steps of metatranscriptomic analyses. [file 42523_2023_246_MOESM1_ESM.tiff]

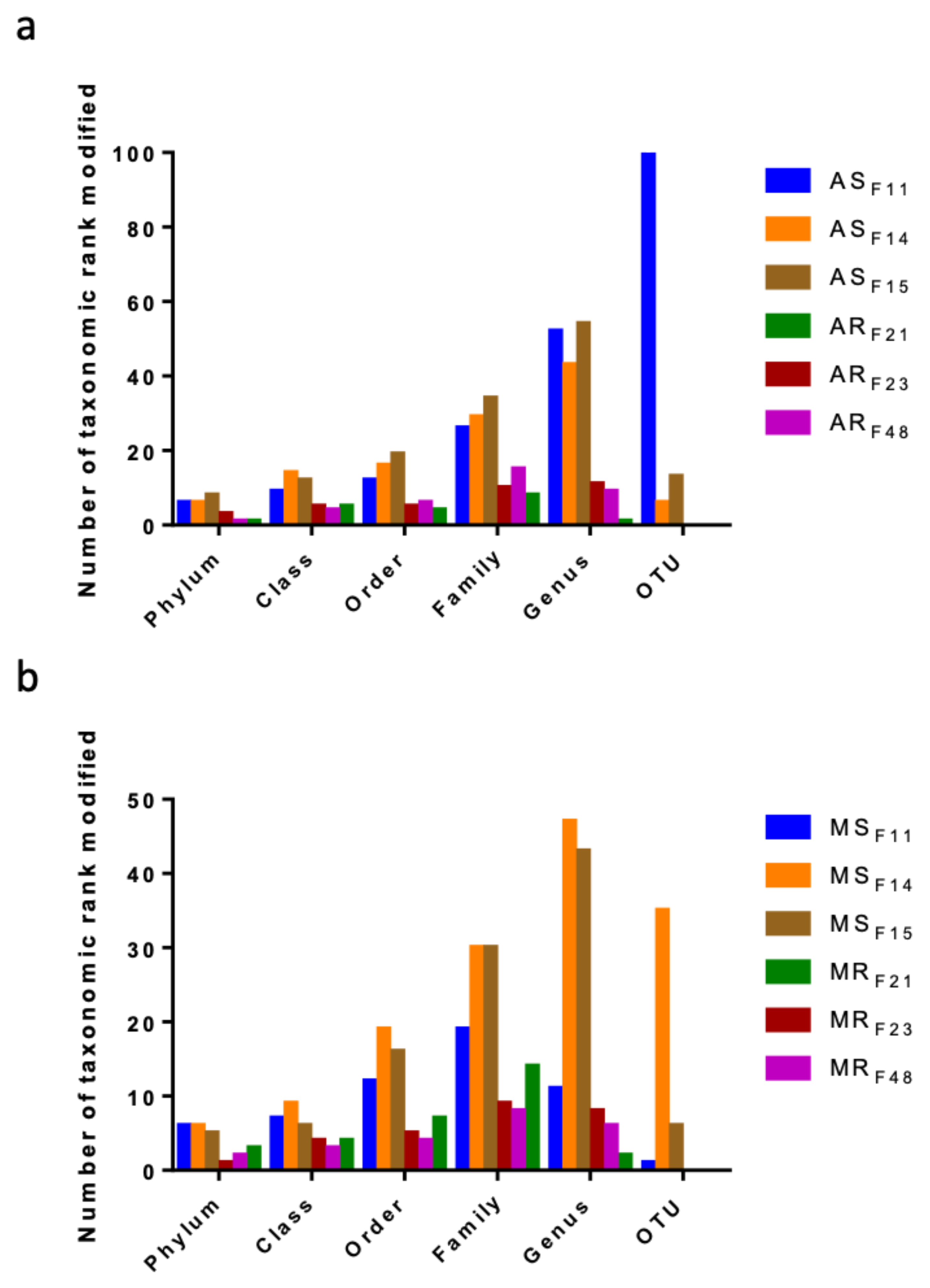

Supplement: Supplementary file 2 — Additional file 2. Figure S2. Microbiota modification as analysed using 16S rRNA metabarcoding in susceptible and resistant oyster families confronted with two different infectious environments. Susceptible oyster familiesand resistant oyster familiesconfronted withAtlantic orMediterranean infectious environments. Significant changes in abundancebetween the initial and the final time point of the infection were much greater for each taxonomic rankfor susceptible oyster families than for resistant oyster families. Data for ASF11 and ARF21 were extracted from [21]. [file 42523_2023_246_MOESM2_ESM.tiff]

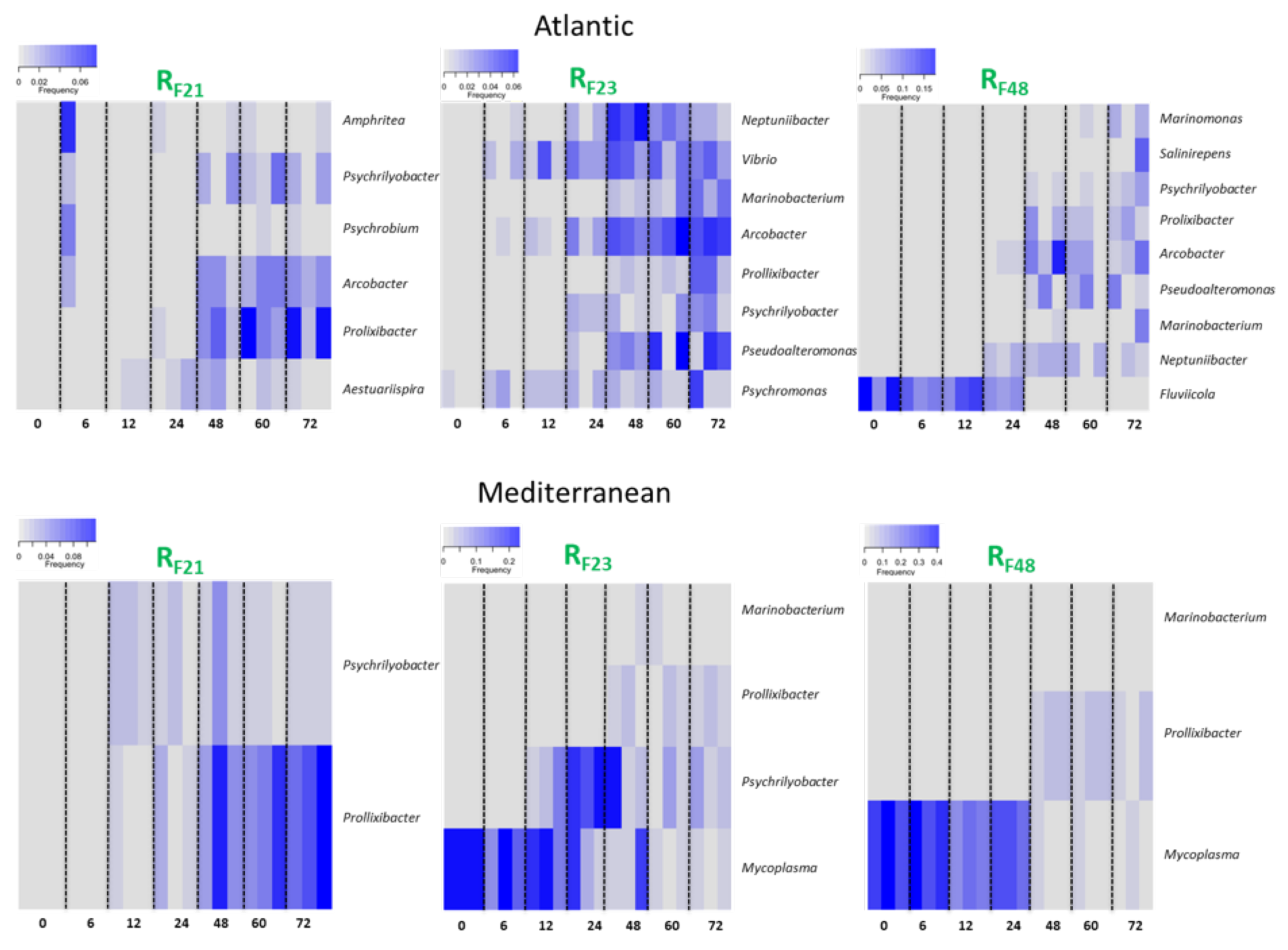

Supplement: Supplementary file 3 — Additional file 3. Figure S3. Heatmaps of bacterial genera that changed significantly in abundance over the course of infection in resistant oystersin the Atlantic and Mediterranean infectious environments. Analyses were performed at the genus level. Only genera with a relative proportion greater than 2% in at least one sample are shown. Increased color intensityindicates increased relative abundance of the genus. [file 42523_2023_246_MOESM3_ESM.tiff]

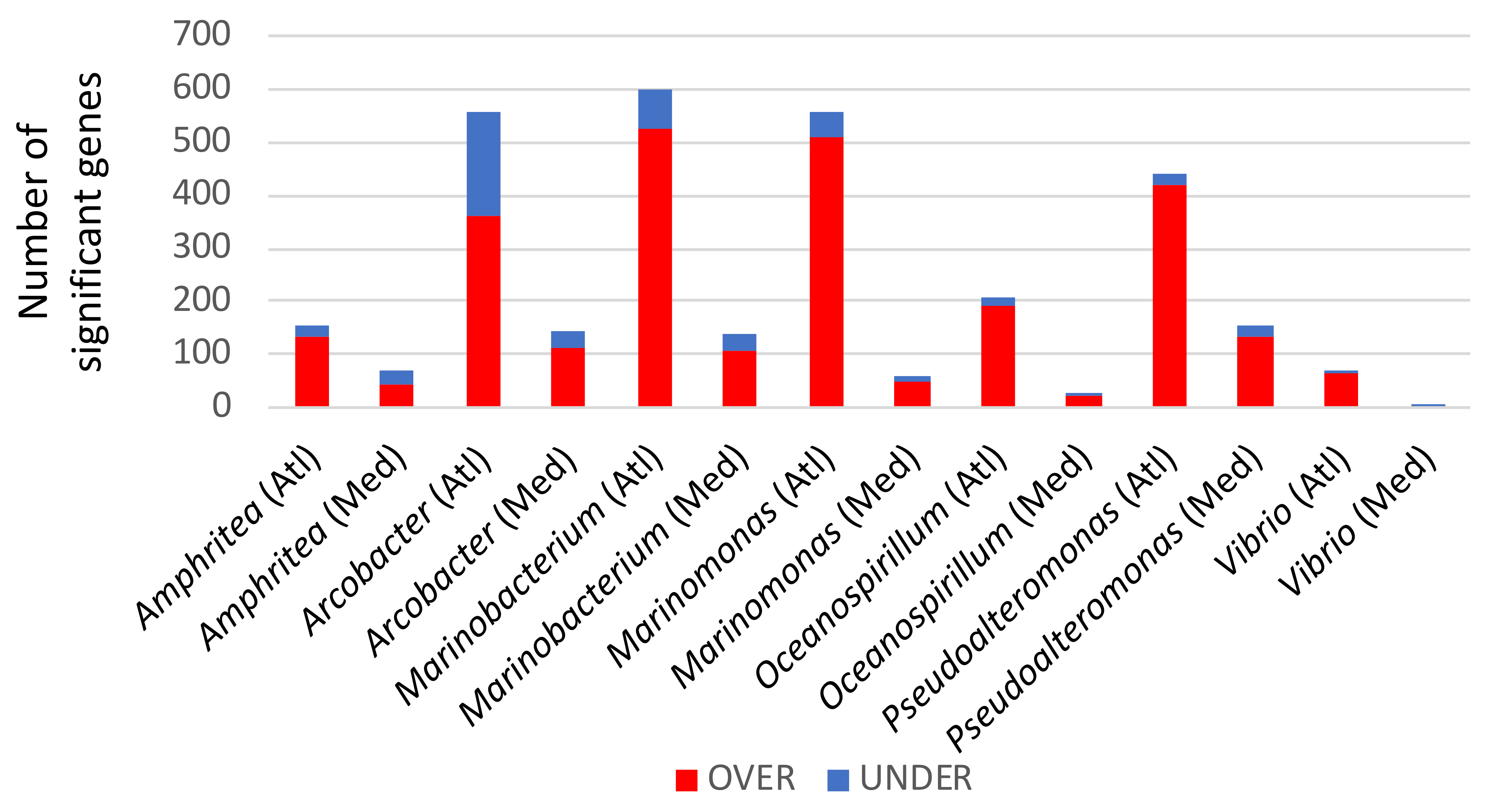

Supplement: Supplementary file 4 — Additional file 4. Figure S4. Number of significant over- and underexpressed genes in each genus and each infectious environment. [file 42523_2023_246_MOESM4_ESM.tiff]

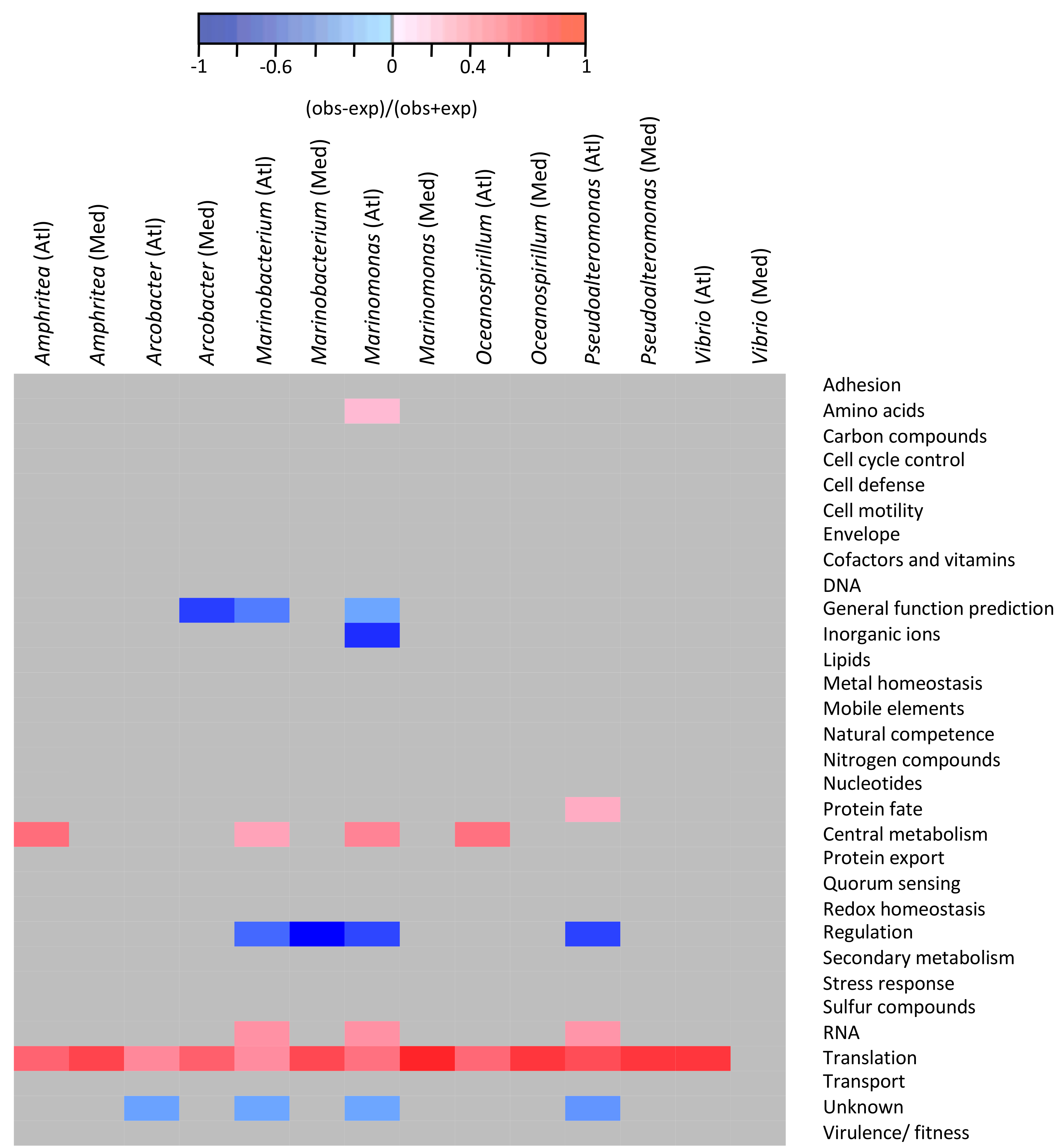

Supplement: Supplementary file 5 — Additional file 5. Figure S5. Enrichment analysis of significant overexpressed bacterial genes in the 31 functional categories. Graded colorsare used to represent the observed over expected values, and indicate under- to overrepresentation, respectively. Grey cells indicate not significant categories. [file 42523_2023_246_MOESM5_ESM.tiff]

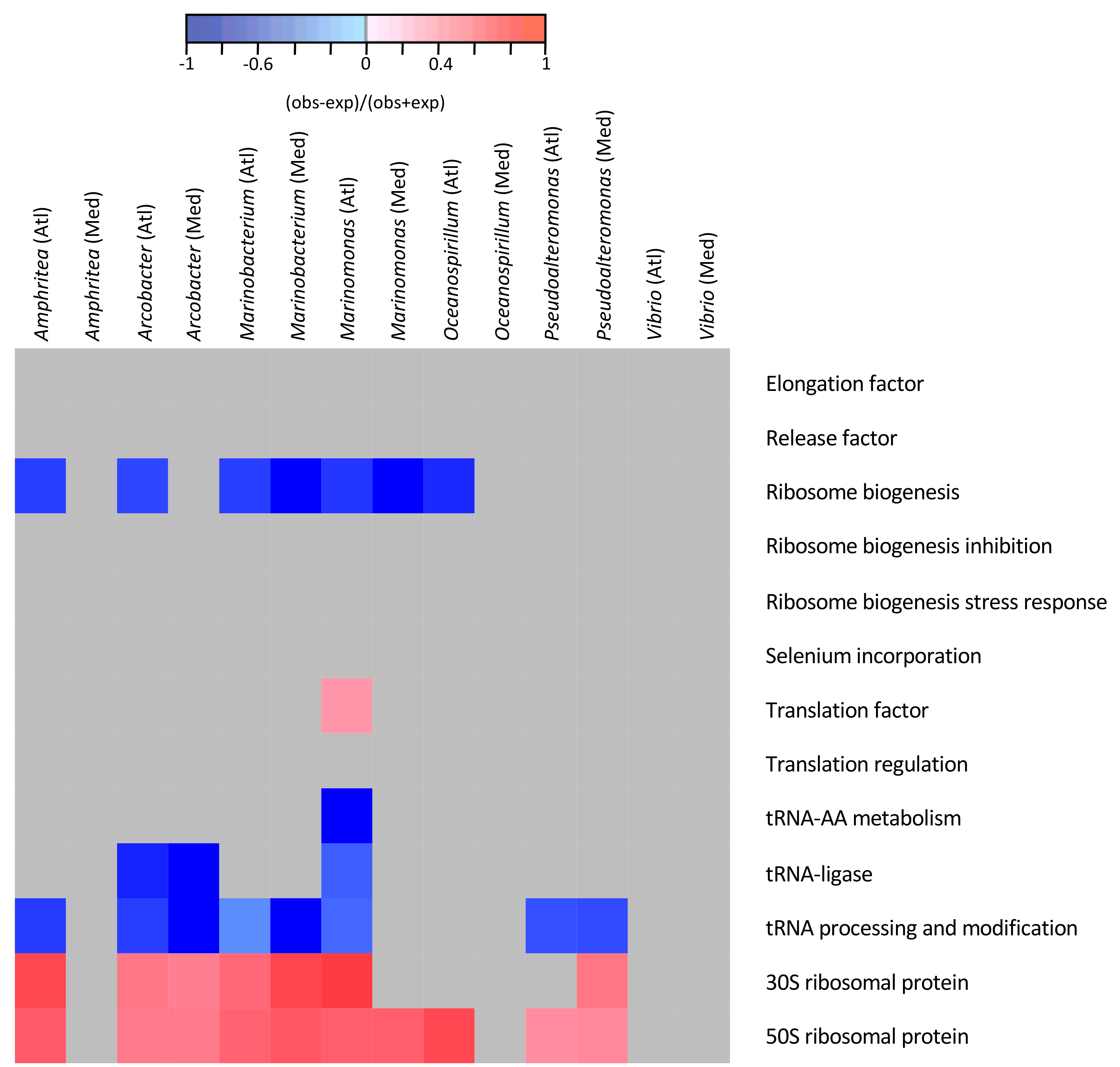

Supplement: Supplementary file 6 — Additional file 6. Figure S6. Enrichment analysis of significant overexpressed bacterial genes within the functional category of translation. Graded colorsare used to represent the observed over expected values, and indicate under- to overrepresentation, respectively. Grey cells indicate not significant subcategories. [file 42523_2023_246_MOESM6_ESM.tiff]

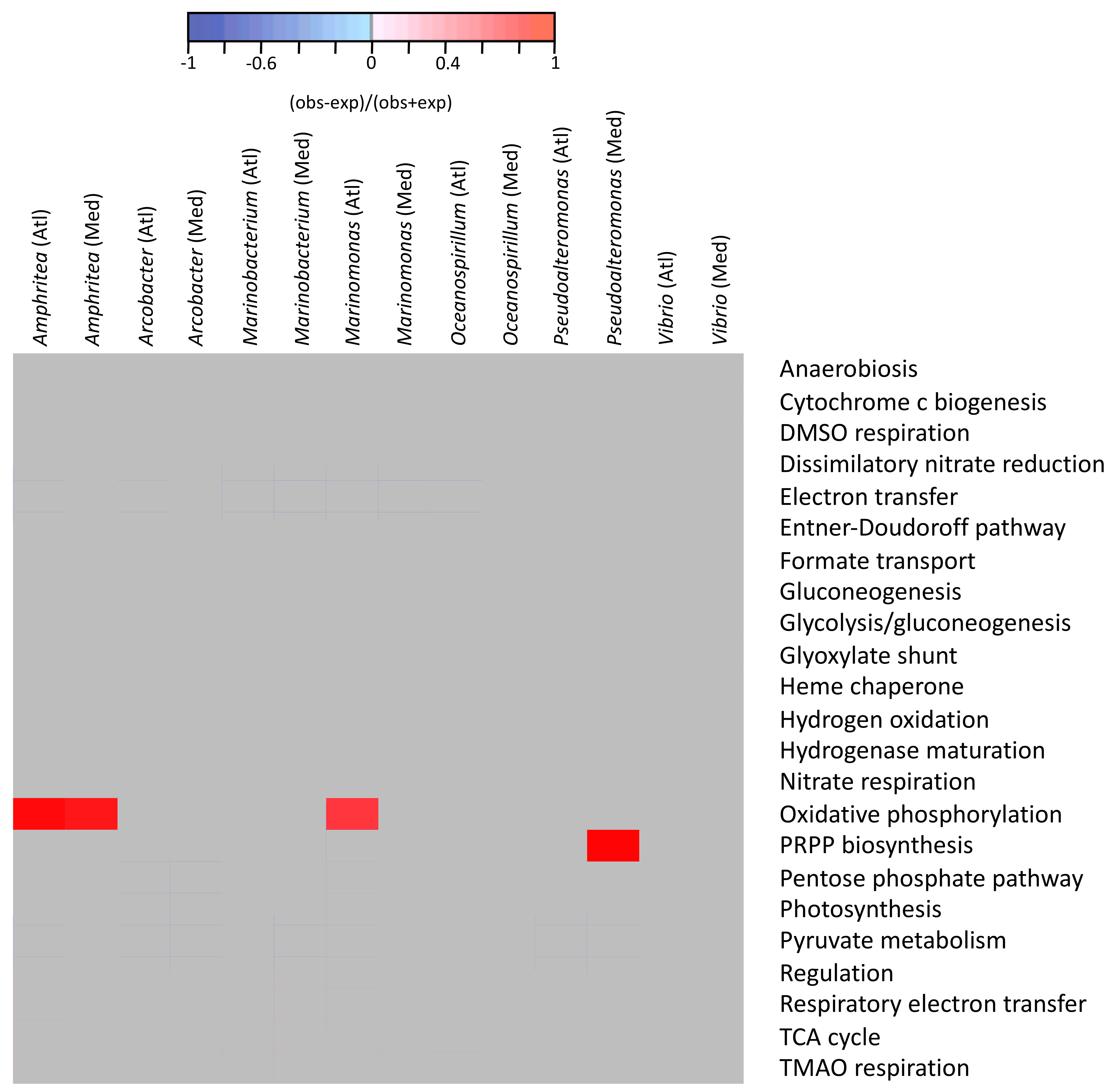

Supplement: Supplementary file 7 — Additional file 7. Figure S7. Enrichment analysis of significant overexpressed bacterial genes within the functional category of central metabolism. Graded colorsare used to represent the observed over expected values, and indicate under- to overrepresentation, respectively. Grey cells indicate not significant subcategories. [file 42523_2023_246_MOESM7_ESM.tiff]

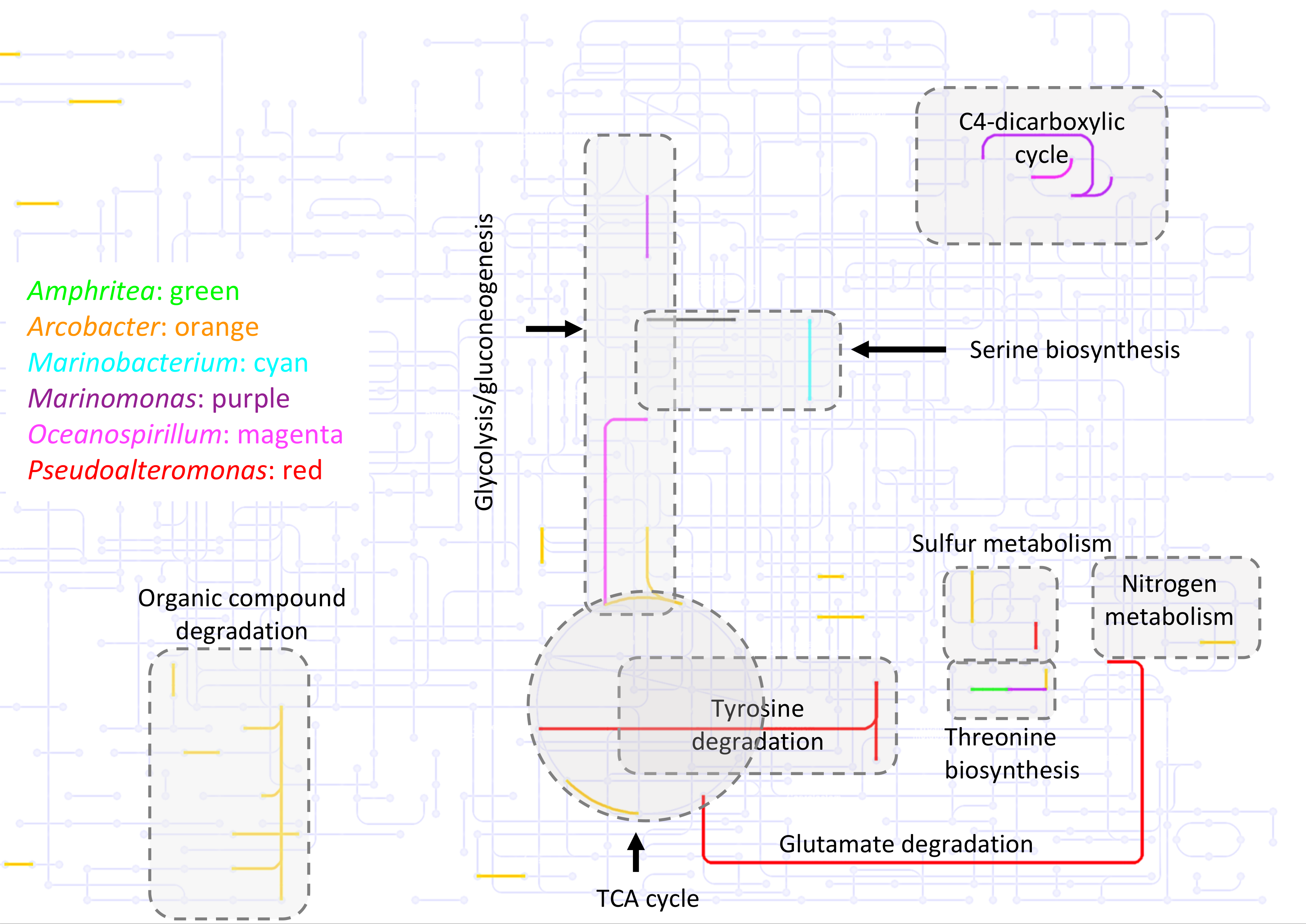

Supplement: Supplementary file 8 — Additional file 8. Figure S8. Decreased expression pathways of the pathobiota metabolism between T0 and the onset of oyster mortality and the contribution of each bacterial genus. EC numbers of differentially underexpressed genes involved in bacterial metabolismwere mapped on KEGG metabolic map 01120using a color code for each genus. KEGG pathways are indicated by shaded boxes. Pathways common to two genera or more are in black. Red arrows indicate the pathway corresponding to neoglucogenesis. Note that not all relevant pathways are represented on this mapwhich was chosen for the sake of clarity. [file 42523_2023_246_MOESM8_ESM.tiff]
